# *N*-(1-Acryloyl-2,2,6,6-tetra­methyl­piperidin-4-yl)acryl­amide

**DOI:** 10.1107/S1600536811042693

**Published:** 2011-10-22

**Authors:** Shailesh K. Goswami, Lyall R. Hanton, C. John McAdam, Stephen C. Moratti, Jim Simpson

**Affiliations:** a Department of Chemistry, University of Otago, PO Box 56, Dunedin, New Zealand

## Abstract

The title compound, C_15_H_24_N_2_O_2_, crystallizes with two unique mol­ecules, (I) and (II), in the asymmetric unit, differing in the orientation of the acryloyl units with respect to the piperidine rings. The acryl­amide units are essentially planar in both mol­ecules (r.m.s. deviations = 0.042 and 0.024 Å, respectively), as are the C_3_N chains of the acryloyl units. The carbonyl O atoms of the acryloyl systems lie significantly out of these planes, *viz.* by  −0.171 (9) Å for molecule (I) and by 0.33 (1) Å for molecule (II). The acryl­amide and acryloyl planes are inclined at 68.7 (4)° and 59.8 (3)° in the two mol­ecules. The piperidine rings each adopt twist boat conformations. In the crystal, strong N—H⋯O hydrogen bonds link the mol­ecules into zigzag *C*(4) chains along *b*. Additional C—H⋯O contacts result in the formation of stacks along *a*.

## Related literature

For the synthesis and applications, see: Murayama & Morimura (1971 ▶); Matsui *et al.* (1972 ▶). Very few structures of compounds similar to the title compound have been reported previously. The most closely related 2,2,6,6-tetra­methyl­piperidine structures are both nitroxide radicals but also have acryl­amide substituents in the 4-position, see: Duskova *et al.* (2006 ▶); Qiu *et al.* (2009 ▶). For other related 2,2,6,6-tetra­methyl­piperidine structures, see: Cygler, Dobrynin *et al.* (1980 ▶); Cygler, Skarżyński *et al.* (1980 ▶); Cygler, Markowicz *et al.* (1980 ▶); Cygler (1981 ▶). For details of the Cambridge Structural Database, see: Allen (2002 ▶); and for hydrogen-bond motifs, see: Bernstein *et al.* (1995 ▶)
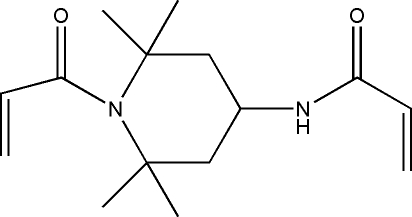


## Experimental

### 

#### Crystal data

C_15_H_24_N_2_O_2_
*M* *_r_* = 264.36
Monoclinic, 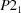

*a* = 7.5810 (4) Å
*b* = 9.2635 (4) Å
*c* = 21.4193 (9) Å
β = 91.612 (2)°
*V* = 1503.61 (12) Å^3^
*Z* = 4
Mo *K*α radiation
μ = 0.08 mm^−1^
*T* = 91 K
0.65 × 0.24 × 0.14 mm

#### Data collection

Bruker APEXII CCD area detector diffractometer
Absorption correction: multi-scan (*SADABS*; Bruker, 2009 ▶) *T*_min_ = 0.690, *T*_max_ = 0.744
9141 measured reflections
2252 independent reflections
2169 reflections with *I* > 2σ(*I*)
*R*_int_ = 0.040
θ_max_ = 18.6°

#### Refinement

*R*[*F* ^2^ > 2σ(*F* ^2^)] = 0.030
*wR*(*F* ^2^) = 0.069
*S* = 1.12
2252 reflections
357 parameters
1 restraint
H atoms treated by a mixture of independent and constrained refinement
Δρ_max_ = 0.10 e Å^−3^
Δρ_min_ = −0.12 e Å^−3^

### 

Data collection: *APEX2* (Bruker 2009 ▶); cell refinement: *APEX2* and *SAINT* (Bruker 2009 ▶); data reduction: *SAINT*; program(s) used to solve structure: *SHELXS97* (Sheldrick, 2008 ▶), *TITAN2000* (Hunter & Simpson, 1999 ▶); program(s) used to refine structure: *SHELXL97* (Sheldrick, 2008 ▶), *TITAN2000*; molecular graphics: *SHELXTL* (Sheldrick, 2008 ▶), *Mercury* (Macrae *et al.*, 2008 ▶); software used to prepare material for publication: *SHELXL97*, *enCIFer* (Allen *et al.*, 2004 ▶), *PLATON* (Spek, 2009 ▶), *publCIF* (Westrip, 2010 ▶).

## Supplementary Material

Crystal structure: contains datablock(s) global, I. DOI: 10.1107/S1600536811042693/cv5174sup1.cif

Structure factors: contains datablock(s) I. DOI: 10.1107/S1600536811042693/cv5174Isup2.hkl

Supplementary material file. DOI: 10.1107/S1600536811042693/cv5174Isup3.cml

Additional supplementary materials:  crystallographic information; 3D view; checkCIF report

## Figures and Tables

**Table 1:** Hydrogen-bond geometry (Å, °)

*D*—H⋯*A*	*D*—H	H⋯*A*	*D*⋯*A*	*D*—H⋯*A*
N22—H22*N*⋯O17	0.87 (3)	2.02 (3)	2.888 (4)	171 (3)
N12—H12*N*⋯O27^i^	0.87 (3)	1.98 (3)	2.841 (4)	177 (3)
C15—H15*B*⋯O13^ii^	0.99	2.67	3.613 (4)	159

Symmetry codes: (i) 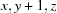; (ii) 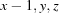.

**Fig. 1.**
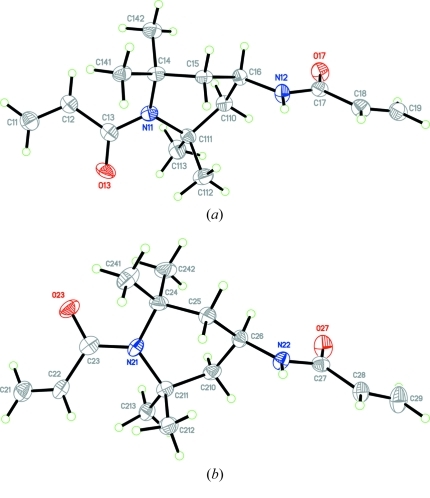
The structure of independent molecules I (a) and II (b) of the title compound with displacement ellipsoids drawn at the 50% probability level.

**Fig. 2.**
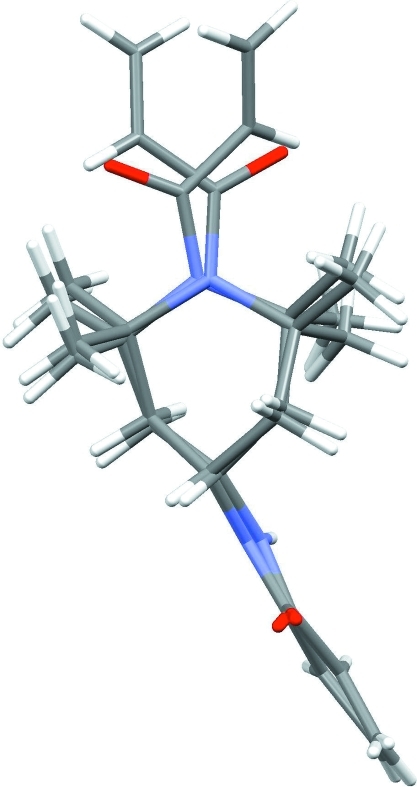
An overlay of the two unique molecules 1 & 2. The r.m.s. deviation is 0.90 Å.

**Fig. 3.**
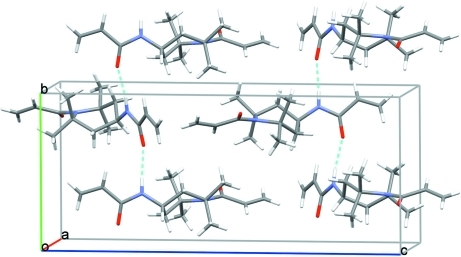
Zigzag C(4) chains along b formed by N–H···O hydrogen bonds.

**Fig. 4.**
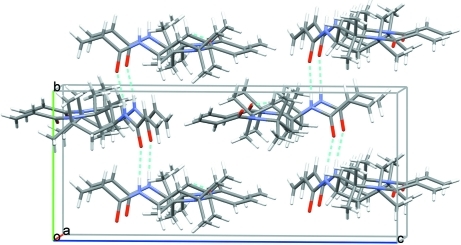
Crystal packing of molecules of the title compound.

## supplementary crystallographic information

### Comment

The title compound has been used for stabilization of synthetic polymers against photo and thermal deterioration (Murayama & Morimura, 1971; Matsui *et al.*, 1972). Our synthesis and interest relates to its use as a cross-linker in our work with electroactive polymers. The title compound, crystallizes with two unique molecules (I), Fig. 1a, and (II), Fig. 1b, linked in the asymmetric unit by an N—H···O hydrogen bond. Overlaying the two molecules in Mercury (Macrae *et al.*, 2008), Fig 2, gives an r.m.s. deviation of 0.90 Å. The major differences lie in the orientation of the acryloyl units with respect to the piperidine rings, exemplified by the torsion angles C14–N11–C13–O13, -151.1 (3)° for (I) and C24–N21–C23–O23, -20.0 (5)° for (II). Each piperidine ring adopts a twist boat conformation with the acrylamide substituents equatorial. The N12–C17(O17)–C18–C19 and N22–C27(O27)–C28–C29 acrylamide substituents are planar, r.m.s. deviations 0.043 Å and 0.024 Å respectively as are the N11–C13–C12–C11 and N21–C23–C22–C21 chains of the acryloyl units with deviations 0.030 and 0.095 Å.

A search of the Cambridge Database (version 5.32, November 2010, with 5 updates; Allen, 2002) for 2,2,6,6-tetramethylpiperidine residues with C substituents on both the 1 and 4-positions revealed only 4 piperidinol derivatives (Cygler, Dobrynin*et al.*, 1980; Cygler, Skarżyński *et al.*, 1980; Cygler, Markowicz *et al.*, 1980; Cygler, 1981). Only two structures with acrylamide substituents in the 4-position were found (Duskova *et al.*, 2006); Qiu *et al.*, 2009), both involving nitroxide radicals. Bond distances and angles in the acrylamide segments of these structures compare well with those reported here.

In the crystal, C(4) chains (Bernstein *et al.*, 1995) of molecules form along the *b* axis linked by strong intermolecular N—H···O hydrogen bonds (Table 1, Fig 3). Additional C15–H15B···O13 contacts (Table 1) cause the chains to form stacks along the *a* axis (Fig. 4).

### Experimental

The title compound was synthesized in a manner similar to that previously reported (Murayama & Morimura, 1971). Following purification by column chromatography on silica gel and recrystallization, X-ray quality crystals of the title compound, *N*-(1-acryloyl-2,2,6,6-tetramethylpiperidin-4-yl)acrylamide were obtained from a CDCl_3_ solution layered with ethanol. *M*.p. 113°C. ^1^H NMR (400 MHz, CDCl_3_): 6.53 & 6.28 [2 × (1*H*, dd, J = 1, 18 Hz, *trans-* =CH_2_)], 6.10 (2*H*, m, 2 × –CH=), 5.9 (1*H*, bs, amide NH), 5.64 & 5.49 [2 × (1*H*, dd, J = 1, 10 Hz, *cis-* =CH_2_)], 4.40 (1*H*, m, pip CH), 2.25 & 1.77 [2 × (2*H*, dd, J = 8, 16 Hz, pip CH_2_)], 1.53 & 1.49 [2 × (6*H*, s, CH_3_)]. ^13^C NMR (500 MHz, CDCl_3_): 169.9, 164.9, 135.5, 130.6, 126.7, 124.0, 56.1, 44.1, 40.3, 31.2, 29.7.

### Refinement

Crystals were very weakly diffracting and data of reasonable intensity could not be obtained beyond θ = 18.5°. This also contributes to the relatively poor data/parameter ratio observed for this refinement. The absolute structure could not be determined reliably due to the absence of significant anomalous scattering effects. The Flack parameter is not therefore reported. One reflection, signalled in CheckCIF as likely to be affected by the beamstop, was omitted from the final refinement cycles.

The H atoms bound to the amide N atoms were found in a difference Fourier map and their coordinates refined with *U*_iso_=1.2*U*_eq_ (N). All H-atoms bound to carbon were refined using a riding model with d(C—H) = 0.95 Å, for aromatic, 0.99Å for methylene and 1.00 for methine H atoms. The CH_2_ and C–H H atoms of the acryloyl and acrylamide units had d(C—H) = 0.95 Å. All of these had *U*_iso_=1.2*U*_eq_ (C). For the methyl H atoms d(C—H) = 0.98 Å with *U*_iso_ = 1.5*U*_eq_ (C).

### Crystal data

**Table:**

C_15_H_24_N_2_O_2_	*F*(000) = 576
*M**_r_* = 264.36	*D*_x_ = 1.168 Mg m^−^^3^
Monoclinic, *P*2_1_	Mo *K*α radiation, λ = 0.71073 Å
Hall symbol: P 2yb	Cell parameters from 3272 reflections
*a* = 7.5810 (4) Å	θ = 2.4–18.6°
*b* = 9.2635 (4) Å	µ = 0.08 mm^−^^1^
*c* = 21.4193 (9) Å	*T* = 91 K
β = 91.612 (2)°	Block, colourless
*V* = 1503.61 (12) Å^3^	0.65 × 0.24 × 0.14 mm
*Z* = 4	

### Data collection

**Table:**

Bruker APEXII CCD area detector diffractometer	2252 independent reflections
Radiation source: fine-focus sealed tube	2169 reflections with *I* > 2σ(*I*)
graphite	*R*_int_ = 0.040
φ and ω scans	θ_max_ = 18.6°, θ_min_ = 1.0°
Absorption correction: multi-scan (*SADABS*; Bruker, 2009)	*h* = −6→6
*T*_min_ = 0.690, *T*_max_ = 0.744	*k* = −8→8
9141 measured reflections	*l* = −19→19

### Refinement

**Table:**

Refinement on *F*^2^	Primary atom site location: structure-invariant direct methods
Least-squares matrix: full	Secondary atom site location: difference Fourier map
*R*[*F*^2^ > 2σ(*F*^2^)] = 0.030	Hydrogen site location: inferred from neighbouring sites
*wR*(*F*^2^) = 0.069	H atoms treated by a mixture of independent and constrained refinement
*S* = 1.12	*w* = 1/[σ^2^(*F*_o_^2^) + (0.031*P*)^2^ + 0.2286*P*] where *P* = (*F*_o_^2^ + 2*F*_c_^2^)/3
2252 reflections	(Δ/σ)_max_ < 0.001
357 parameters	Δρ_max_ = 0.10 e Å^−^^3^
1 restraint	Δρ_min_ = −0.12 e Å^−^^3^

### Special details

**Table:**

Geometry. All e.s.d.'s (except the e.s.d. in the dihedral angle between two l.s. planes) are estimated using the full covariance matrix. The cell e.s.d.'s are taken into account individually in the estimation of e.s.d.'s in distances, angles and torsion angles; correlations between e.s.d.'s in cell parameters are only used when they are defined by crystal symmetry. An approximate (isotropic) treatment of cell e.s.d.'s is used for estimating e.s.d.'s involving l.s. planes.
Refinement. Refinement of *F*^2^ against ALL reflections. The weighted *R*-factor *wR* and goodness of fit *S* are based on *F*^2^, conventional *R*-factors *R* are based on *F*, with *F* set to zero for negative *F*^2^. The threshold expression of *F*^2^ > σ(*F*^2^) is used only for calculating *R*-factors(gt) *etc*. and is not relevant to the choice of reflections for refinement. *R*-factors based on *F*^2^ are statistically about twice as large as those based on *F*, and *R*- factors based on ALL data will be even larger.

### Fractional atomic coordinates and isotropic or equivalent isotropic displacement parameters (Å^2^)

**Table:**

	*x*	*y*	*z*	*U*_iso_*/*U*_eq_	
C11	0.6512 (5)	0.7963 (4)	0.58401 (18)	0.0351 (10)	
H11A	0.7574	0.8454	0.5748	0.042*	
H11B	0.6307	0.7656	0.6255	0.042*	
C12	0.5323 (5)	0.7710 (3)	0.53929 (17)	0.0266 (9)	
H12	0.4266	0.7219	0.5490	0.032*	
C13	0.5611 (6)	0.8182 (4)	0.47352 (16)	0.0254 (9)	
O13	0.7087 (4)	0.8616 (2)	0.45947 (10)	0.0331 (6)	
N11	0.4267 (4)	0.8008 (3)	0.42980 (12)	0.0235 (7)	
C14	0.2342 (4)	0.8154 (3)	0.44501 (15)	0.0223 (9)	
C141	0.2071 (4)	0.9268 (3)	0.49667 (14)	0.0264 (9)	
H14A	0.2608	0.8915	0.5359	0.040*	
H14B	0.0805	0.9421	0.5020	0.040*	
H14C	0.2626	1.0182	0.4852	0.040*	
C142	0.1516 (4)	0.6705 (4)	0.46339 (15)	0.0315 (10)	
H14D	0.1660	0.6005	0.4296	0.047*	
H14E	0.0256	0.6842	0.4707	0.047*	
H14F	0.2104	0.6343	0.5016	0.047*	
C15	0.1346 (4)	0.8760 (3)	0.38813 (14)	0.0215 (9)	
H15A	0.1841	0.9718	0.3780	0.026*	
H15B	0.0094	0.8899	0.3986	0.026*	
C16	0.1433 (4)	0.7795 (3)	0.33054 (15)	0.0216 (9)	
H16	0.0558	0.6996	0.3349	0.026*	
N12	0.0923 (4)	0.8644 (3)	0.27579 (14)	0.0248 (8)	
H12N	0.091 (4)	0.958 (4)	0.2776 (15)	0.030*	
C17	0.0539 (4)	0.8041 (4)	0.22070 (18)	0.0244 (9)	
O17	0.0605 (3)	0.6724 (3)	0.21106 (10)	0.0312 (6)	
C18	0.0062 (4)	0.9074 (4)	0.17059 (18)	0.0293 (10)	
H18	−0.0116	1.0059	0.1810	0.035*	
C19	−0.0126 (4)	0.8670 (4)	0.11159 (19)	0.0351 (10)	
H19A	0.0048	0.7689	0.1004	0.042*	
H19B	−0.0434	0.9361	0.0804	0.042*	
C110	0.3267 (4)	0.7126 (4)	0.32590 (15)	0.0255 (9)	
H11C	0.3572	0.7099	0.2813	0.031*	
H11D	0.3204	0.6114	0.3406	0.031*	
C111	0.4782 (4)	0.7879 (4)	0.36235 (15)	0.0231 (9)	
C112	0.5237 (5)	0.9342 (4)	0.33346 (15)	0.0338 (10)	
H11E	0.4170	0.9936	0.3299	0.051*	
H11F	0.5712	0.9190	0.2919	0.051*	
H11G	0.6121	0.9833	0.3601	0.051*	
C113	0.6372 (4)	0.6870 (4)	0.35721 (15)	0.0354 (10)	
H11H	0.7445	0.7377	0.3713	0.053*	
H11I	0.6489	0.6571	0.3136	0.053*	
H11J	0.6199	0.6016	0.3834	0.053*	
C21	0.5528 (5)	0.3323 (4)	−0.09531 (17)	0.0352 (10)	
H21A	0.6555	0.2736	−0.0956	0.042*	
H21B	0.4956	0.3596	−0.1336	0.042*	
C22	0.4897 (4)	0.3759 (4)	−0.04192 (17)	0.0272 (9)	
H22	0.3870	0.4345	−0.0420	0.033*	
C23	0.5771 (6)	0.3345 (3)	0.01908 (17)	0.0268 (9)	
O23	0.7355 (4)	0.3018 (3)	0.01962 (10)	0.0348 (6)	
N21	0.4829 (3)	0.3447 (3)	0.07266 (13)	0.0230 (7)	
C24	0.5850 (4)	0.3639 (4)	0.13359 (15)	0.0275 (9)	
C241	0.7226 (5)	0.4842 (4)	0.12734 (17)	0.0388 (11)	
H24A	0.8099	0.4554	0.0969	0.058*	
H24B	0.7818	0.5010	0.1679	0.058*	
H24C	0.6639	0.5731	0.1132	0.058*	
C242	0.6742 (5)	0.2224 (4)	0.15384 (16)	0.0345 (10)	
H24D	0.5852	0.1461	0.1564	0.052*	
H24E	0.7326	0.2354	0.1949	0.052*	
H24F	0.7620	0.1952	0.1232	0.052*	
C25	0.4583 (4)	0.4172 (4)	0.18298 (14)	0.0258 (9)	
H25A	0.5261	0.4365	0.2223	0.031*	
H25B	0.4047	0.5093	0.1687	0.031*	
C26	0.3114 (4)	0.3095 (4)	0.19614 (15)	0.0238 (9)	
H26	0.3634	0.2300	0.2223	0.029*	
N22	0.1734 (4)	0.3780 (3)	0.23152 (14)	0.0229 (7)	
H22N	0.146 (4)	0.469 (4)	0.2289 (14)	0.028*	
C27	0.0742 (5)	0.3013 (5)	0.27007 (16)	0.0239 (9)	
O27	0.1022 (3)	0.1708 (3)	0.28043 (10)	0.0337 (7)	
C28	−0.0730 (5)	0.3785 (4)	0.29870 (15)	0.0259 (9)	
H28	−0.0865	0.4792	0.2918	0.031*	
C29	−0.1860 (5)	0.3102 (4)	0.33360 (16)	0.0399 (10)	
H29A	−0.1736	0.2095	0.3407	0.048*	
H29B	−0.2802	0.3617	0.3517	0.048*	
C210	0.2424 (4)	0.2433 (3)	0.13444 (14)	0.0267 (10)	
H21C	0.2882	0.1435	0.1322	0.032*	
H21D	0.1124	0.2365	0.1364	0.032*	
C211	0.2858 (4)	0.3195 (3)	0.07325 (15)	0.0246 (9)	
C212	0.1822 (4)	0.4611 (3)	0.06547 (15)	0.0289 (9)	
H21E	0.1859	0.5143	0.1050	0.043*	
H21F	0.0593	0.4395	0.0535	0.043*	
H21G	0.2353	0.5200	0.0329	0.043*	
C213	0.2268 (5)	0.2124 (4)	0.02233 (15)	0.0297 (9)	
H21H	0.2345	0.2584	−0.0187	0.044*	
H21I	0.1047	0.1829	0.0291	0.044*	
H21J	0.3036	0.1273	0.0241	0.044*	

### Atomic displacement parameters (Å^2^)

**Table:**

	*U*^11^	*U*^22^	*U*^33^	*U*^12^	*U*^13^	*U*^23^
C11	0.040 (3)	0.028 (2)	0.037 (3)	−0.002 (2)	0.003 (2)	−0.004 (2)
C12	0.026 (3)	0.023 (2)	0.030 (3)	0.0039 (18)	−0.001 (2)	−0.0063 (19)
C13	0.026 (3)	0.020 (2)	0.030 (3)	0.005 (2)	0.002 (2)	−0.009 (2)
O13	0.0183 (16)	0.0412 (15)	0.0399 (16)	−0.0068 (14)	0.0042 (13)	−0.0083 (12)
N11	0.022 (2)	0.0237 (16)	0.025 (2)	0.0030 (15)	0.0058 (17)	−0.0015 (15)
C14	0.021 (2)	0.019 (2)	0.027 (2)	0.0028 (19)	0.0062 (19)	−0.001 (2)
C141	0.025 (2)	0.024 (2)	0.030 (2)	0.0001 (17)	0.0034 (17)	0.005 (2)
C142	0.029 (3)	0.027 (2)	0.038 (2)	−0.004 (2)	0.0021 (18)	0.003 (2)
C15	0.016 (2)	0.0180 (19)	0.030 (2)	0.0024 (18)	0.0023 (17)	0.005 (2)
C16	0.025 (2)	0.015 (2)	0.024 (2)	0.0013 (18)	−0.0035 (17)	0.002 (2)
N12	0.035 (2)	0.0128 (16)	0.026 (2)	−0.0007 (16)	−0.0067 (15)	−0.003 (2)
C17	0.024 (2)	0.013 (3)	0.036 (3)	0.001 (2)	−0.0020 (19)	0.006 (3)
O17	0.0376 (17)	0.0171 (17)	0.0384 (15)	0.0001 (13)	−0.0071 (12)	0.0008 (14)
C18	0.031 (2)	0.019 (2)	0.038 (3)	−0.0008 (18)	−0.0008 (19)	−0.003 (2)
C19	0.032 (2)	0.027 (2)	0.047 (3)	0.000 (2)	−0.009 (2)	0.011 (2)
C110	0.025 (3)	0.024 (2)	0.028 (2)	0.003 (2)	0.0019 (18)	0.0006 (18)
C111	0.018 (2)	0.025 (2)	0.026 (2)	0.000 (2)	0.0005 (18)	−0.0031 (19)
C112	0.039 (3)	0.031 (2)	0.032 (2)	−0.006 (2)	0.0103 (19)	−0.002 (2)
C113	0.030 (3)	0.044 (3)	0.032 (2)	0.005 (2)	0.0034 (18)	−0.009 (2)
C21	0.030 (2)	0.035 (2)	0.041 (3)	−0.0057 (19)	0.004 (2)	0.004 (2)
C22	0.021 (2)	0.027 (2)	0.034 (3)	−0.0027 (19)	0.009 (2)	−0.002 (2)
C23	0.027 (3)	0.018 (2)	0.035 (3)	−0.002 (2)	0.002 (2)	−0.0048 (19)
O23	0.0189 (17)	0.0442 (16)	0.0414 (16)	0.0068 (14)	0.0054 (13)	−0.0057 (13)
N21	0.019 (2)	0.0218 (18)	0.029 (2)	0.0010 (14)	0.0065 (17)	−0.0030 (14)
C24	0.022 (2)	0.036 (2)	0.024 (2)	0.004 (2)	−0.0042 (19)	−0.0069 (19)
C241	0.025 (3)	0.043 (2)	0.049 (3)	−0.005 (2)	0.007 (2)	−0.007 (2)
C242	0.027 (2)	0.036 (3)	0.040 (2)	0.013 (2)	−0.0052 (19)	−0.003 (2)
C25	0.025 (2)	0.028 (2)	0.025 (2)	0.001 (2)	0.0008 (18)	−0.0010 (19)
C26	0.024 (2)	0.022 (2)	0.025 (2)	0.001 (2)	0.0038 (18)	0.0012 (19)
N22	0.0227 (19)	0.0166 (16)	0.0297 (18)	0.0032 (18)	0.0052 (16)	0.0021 (18)
C27	0.025 (3)	0.022 (3)	0.025 (2)	−0.001 (2)	−0.005 (2)	−0.005 (2)
O27	0.0494 (18)	0.0176 (16)	0.0348 (16)	0.0045 (14)	0.0093 (12)	0.0033 (13)
C28	0.031 (3)	0.022 (2)	0.026 (2)	−0.002 (2)	0.005 (2)	0.000 (2)
C29	0.053 (3)	0.031 (2)	0.037 (2)	0.003 (2)	0.017 (2)	−0.004 (2)
C210	0.023 (2)	0.022 (2)	0.036 (2)	0.0053 (17)	0.0010 (19)	0.004 (2)
C211	0.022 (3)	0.024 (2)	0.028 (2)	0.000 (2)	0.0001 (17)	0.001 (2)
C212	0.025 (2)	0.029 (2)	0.032 (2)	0.001 (2)	0.0006 (18)	0.0027 (19)
C213	0.026 (2)	0.033 (2)	0.031 (2)	−0.0056 (19)	0.0042 (18)	0.002 (2)

### Geometric parameters (Å, °)

**Table:**

C11—C12	1.318 (4)	C21—C22	1.315 (4)
C11—H11A	0.9500	C21—H21A	0.9500
C11—H11B	0.9500	C21—H21B	0.9500
C12—C13	1.497 (5)	C22—C23	1.498 (5)
C12—H12	0.9500	C22—H22	0.9500
C13—O13	1.234 (4)	C23—O23	1.238 (4)
C13—N11	1.374 (4)	C23—N21	1.372 (4)
N11—C14	1.510 (4)	N21—C24	1.509 (4)
N11—C111	1.512 (4)	N21—C211	1.513 (4)
C14—C15	1.522 (4)	C24—C25	1.531 (4)
C14—C141	1.531 (4)	C24—C242	1.532 (5)
C14—C142	1.538 (5)	C24—C241	1.535 (5)
C141—H14A	0.9800	C241—H24A	0.9800
C141—H14B	0.9800	C241—H24B	0.9800
C141—H14C	0.9800	C241—H24C	0.9800
C142—H14D	0.9800	C242—H24D	0.9800
C142—H14E	0.9800	C242—H24E	0.9800
C142—H14F	0.9800	C242—H24F	0.9800
C15—C16	1.526 (4)	C25—C26	1.527 (4)
C15—H15A	0.9900	C25—H25A	0.9900
C15—H15B	0.9900	C25—H25B	0.9900
C16—N12	1.455 (4)	C26—N22	1.455 (4)
C16—C110	1.528 (4)	C26—C210	1.535 (4)
C16—H16	1.0000	C26—H26	1.0000
N12—C17	1.330 (4)	N22—C27	1.337 (4)
N12—H12N	0.87 (3)	N22—H22N	0.87 (3)
C17—O17	1.239 (4)	C27—O27	1.246 (4)
C17—C18	1.475 (5)	C27—C28	1.474 (5)
C18—C19	1.322 (4)	C28—C29	1.315 (4)
C18—H18	0.9500	C28—H28	0.9500
C19—H19A	0.9500	C29—H29A	0.9500
C19—H19B	0.9500	C29—H29B	0.9500
C110—C111	1.538 (5)	C210—C211	1.532 (4)
C110—H11C	0.9900	C210—H21C	0.9900
C110—H11D	0.9900	C210—H21D	0.9900
C111—C113	1.532 (4)	C211—C213	1.532 (4)
C111—C112	1.533 (4)	C211—C212	1.535 (4)
C112—H11E	0.9800	C212—H21E	0.9800
C112—H11F	0.9800	C212—H21F	0.9800
C112—H11G	0.9800	C212—H21G	0.9800
C113—H11H	0.9800	C213—H21H	0.9800
C113—H11I	0.9800	C213—H21I	0.9800
C113—H11J	0.9800	C213—H21J	0.9800
			
C12—C11—H11A	120.0	C22—C21—H21A	120.0
C12—C11—H11B	120.0	C22—C21—H21B	120.0
H11A—C11—H11B	120.0	H21A—C21—H21B	120.0
C11—C12—C13	121.2 (4)	C21—C22—C23	121.2 (3)
C11—C12—H12	119.4	C21—C22—H22	119.4
C13—C12—H12	119.4	C23—C22—H22	119.4
O13—C13—N11	122.1 (3)	O23—C23—N21	122.4 (3)
O13—C13—C12	118.8 (3)	O23—C23—C22	118.4 (3)
N11—C13—C12	118.9 (4)	N21—C23—C22	119.0 (3)
C13—N11—C14	123.0 (3)	C23—N21—C24	117.7 (3)
C13—N11—C111	117.0 (3)	C23—N21—C211	122.3 (3)
C14—N11—C111	119.4 (3)	C24—N21—C211	119.6 (3)
N11—C14—C15	108.7 (2)	N21—C24—C25	108.6 (3)
N11—C14—C141	111.5 (3)	N21—C24—C242	110.8 (3)
C15—C14—C141	104.9 (2)	C25—C24—C242	111.2 (3)
N11—C14—C142	112.3 (3)	N21—C24—C241	110.1 (3)
C15—C14—C142	109.3 (3)	C25—C24—C241	105.5 (3)
C141—C14—C142	109.9 (2)	C242—C24—C241	110.5 (3)
C14—C141—H14A	109.5	C24—C241—H24A	109.5
C14—C141—H14B	109.5	C24—C241—H24B	109.5
H14A—C141—H14B	109.5	H24A—C241—H24B	109.5
C14—C141—H14C	109.5	C24—C241—H24C	109.5
H14A—C141—H14C	109.5	H24A—C241—H24C	109.5
H14B—C141—H14C	109.5	H24B—C241—H24C	109.5
C14—C142—H14D	109.5	C24—C242—H24D	109.5
C14—C142—H14E	109.5	C24—C242—H24E	109.5
H14D—C142—H14E	109.5	H24D—C242—H24E	109.5
C14—C142—H14F	109.5	C24—C242—H24F	109.5
H14D—C142—H14F	109.5	H24D—C242—H24F	109.5
H14E—C142—H14F	109.5	H24E—C242—H24F	109.5
C14—C15—C16	113.5 (3)	C26—C25—C24	113.1 (3)
C14—C15—H15A	108.9	C26—C25—H25A	109.0
C16—C15—H15A	108.9	C24—C25—H25A	109.0
C14—C15—H15B	108.9	C26—C25—H25B	109.0
C16—C15—H15B	108.9	C24—C25—H25B	109.0
H15A—C15—H15B	107.7	H25A—C25—H25B	107.8
N12—C16—C15	108.6 (3)	N22—C26—C25	110.5 (3)
N12—C16—C110	112.9 (3)	N22—C26—C210	112.9 (3)
C15—C16—C110	110.5 (3)	C25—C26—C210	109.6 (3)
N12—C16—H16	108.3	N22—C26—H26	107.9
C15—C16—H16	108.3	C25—C26—H26	107.9
C110—C16—H16	108.3	C210—C26—H26	107.9
C17—N12—C16	122.3 (3)	C27—N22—C26	121.0 (3)
C17—N12—H12N	117 (2)	C27—N22—H22N	114 (2)
C16—N12—H12N	120 (2)	C26—N22—H22N	124 (2)
O17—C17—N12	123.5 (3)	O27—C27—N22	122.0 (3)
O17—C17—C18	121.9 (4)	O27—C27—C28	121.6 (3)
N12—C17—C18	114.6 (3)	N22—C27—C28	116.4 (4)
C19—C18—C17	122.0 (3)	C29—C28—C27	121.1 (3)
C19—C18—H18	119.0	C29—C28—H28	119.4
C17—C18—H18	119.0	C27—C28—H28	119.4
C18—C19—H19A	120.0	C28—C29—H29A	120.0
C18—C19—H19B	120.0	C28—C29—H29B	120.0
H19A—C19—H19B	120.0	H29A—C29—H29B	120.0
C16—C110—C111	116.8 (3)	C211—C210—C26	118.4 (3)
C16—C110—H11C	108.1	C211—C210—H21C	107.7
C111—C110—H11C	108.1	C26—C210—H21C	107.7
C16—C110—H11D	108.1	C211—C210—H21D	107.7
C111—C110—H11D	108.1	C26—C210—H21D	107.7
H11C—C110—H11D	107.3	H21C—C210—H21D	107.1
N11—C111—C113	110.0 (3)	N21—C211—C213	111.2 (3)
N11—C111—C112	112.5 (3)	N21—C211—C210	108.3 (2)
C113—C111—C112	108.9 (3)	C213—C211—C210	104.2 (2)
N11—C111—C110	108.2 (2)	N21—C211—C212	111.7 (2)
C113—C111—C110	105.3 (3)	C213—C211—C212	109.8 (3)
C112—C111—C110	111.7 (3)	C210—C211—C212	111.4 (3)
C111—C112—H11E	109.5	C211—C212—H21E	109.5
C111—C112—H11F	109.5	C211—C212—H21F	109.5
H11E—C112—H11F	109.5	H21E—C212—H21F	109.5
C111—C112—H11G	109.5	C211—C212—H21G	109.5
H11E—C112—H11G	109.5	H21E—C212—H21G	109.5
H11F—C112—H11G	109.5	H21F—C212—H21G	109.5
C111—C113—H11H	109.5	C211—C213—H21H	109.5
C111—C113—H11I	109.5	C211—C213—H21I	109.5
H11H—C113—H11I	109.5	H21H—C213—H21I	109.5
C111—C113—H11J	109.5	C211—C213—H21J	109.5
H11H—C113—H11J	109.5	H21H—C213—H21J	109.5
H11I—C113—H11J	109.5	H21I—C213—H21J	109.5
			
C11—C12—C13—O13	11.0 (5)	C21—C22—C23—O23	23.7 (5)
C11—C12—C13—N11	−174.2 (3)	C21—C22—C23—N21	−161.1 (3)
O13—C13—N11—C14	−151.1 (3)	O23—C23—N21—C24	19.9 (4)
C12—C13—N11—C14	34.2 (4)	C22—C23—N21—C24	−155.1 (3)
O13—C13—N11—C111	20.0 (5)	O23—C23—N21—C211	−152.6 (3)
C12—C13—N11—C111	−154.7 (3)	C22—C23—N21—C211	32.4 (4)
C13—N11—C14—C15	147.4 (3)	C23—N21—C24—C25	163.3 (3)
C111—N11—C14—C15	−23.4 (4)	C211—N21—C24—C25	−24.0 (4)
C13—N11—C14—C141	32.3 (4)	C23—N21—C24—C242	−74.4 (4)
C111—N11—C14—C141	−138.5 (3)	C211—N21—C24—C242	98.4 (3)
C13—N11—C14—C142	−91.5 (4)	C23—N21—C24—C241	48.2 (4)
C111—N11—C14—C142	97.6 (3)	C211—N21—C24—C241	−139.0 (3)
N11—C14—C15—C16	61.2 (3)	N21—C24—C25—C26	62.6 (3)
C141—C14—C15—C16	−179.5 (3)	C242—C24—C25—C26	−59.6 (4)
C142—C14—C15—C16	−61.8 (3)	C241—C24—C25—C26	−179.4 (3)
C14—C15—C16—N12	−163.1 (3)	C24—C25—C26—N22	−166.5 (3)
C14—C15—C16—C110	−38.8 (4)	C24—C25—C26—C210	−41.5 (4)
C15—C16—N12—C17	−166.6 (3)	C25—C26—N22—C27	−151.7 (3)
C110—C16—N12—C17	70.6 (4)	C210—C26—N22—C27	85.2 (4)
C16—N12—C17—O17	−1.1 (5)	C26—N22—C27—O27	4.6 (5)
C16—N12—C17—C18	−179.6 (3)	C26—N22—C27—C28	−174.1 (3)
O17—C17—C18—C19	−7.7 (5)	O27—C27—C28—C29	−3.8 (5)
N12—C17—C18—C19	170.8 (3)	N22—C27—C28—C29	174.9 (3)
N12—C16—C110—C111	101.7 (3)	N22—C26—C210—C211	106.8 (3)
C15—C16—C110—C111	−20.1 (4)	C25—C26—C210—C211	−16.8 (4)
C13—N11—C111—C113	43.7 (4)	C23—N21—C211—C213	29.6 (4)
C14—N11—C111—C113	−144.8 (3)	C24—N21—C211—C213	−142.8 (3)
C13—N11—C111—C112	−77.8 (4)	C23—N21—C211—C210	143.5 (3)
C14—N11—C111—C112	93.6 (3)	C24—N21—C211—C210	−28.9 (4)
C13—N11—C111—C110	158.3 (3)	C23—N21—C211—C212	−93.4 (3)
C14—N11—C111—C110	−30.3 (4)	C24—N21—C211—C212	94.2 (3)
C16—C110—C111—N11	54.3 (4)	C26—C210—C211—N21	51.8 (4)
C16—C110—C111—C113	171.9 (3)	C26—C210—C211—C213	170.3 (3)
C16—C110—C111—C112	−70.1 (4)	C26—C210—C211—C212	−71.4 (4)

### Hydrogen-bond geometry (Å, °)

**Table:**

*D*—H···*A*	*D*—H	H···*A*	*D*···*A*	*D*—H···*A*
N22—H22N···O17	0.87 (3)	2.02 (3)	2.888 (4)	171 (3)
N12—H12N···O27^i^	0.87 (3)	1.98 (3)	2.841 (4)	177 (3)
C15—H15B···O13^ii^	0.99	2.67	3.613 (4)	159.

Symmetry codes: (i) *x*, *y*+1, *z*; (ii) *x*−1, *y*, *z*.
